# Histological Hyperspectral Breast Cancer Recurrence Database (HistologyHSI-BC Recurrence)

**DOI:** 10.1038/s41597-025-06157-4

**Published:** 2025-11-28

**Authors:** Laura Quintana-Quintana, Esther Sauras-Colón, Alessio Fiorin, Javier Santana-Nunez, Samuel Ortega, Noèlia Gallardo-Borràs, Alba Fischer-Carles, Tábata Sánchez-Alcántara, Himar Fabelo, Laia Adalid-Llansa, Daniel Mata-Cano, Ramon Bosch-Príncep, Marylène Lejeune, Gustavo M. Callico, Carlos López-Pablo

**Affiliations:** 1 Institute for Applied Microelectronics, University of Las Palmas de Gran Canaria, Las Palmas de Gran Canaria, Spain; 2 Department of Pathology, Hospital de Tortosa Verge de la Cinta, Institut Català de la Salut, Tortosa, Spain; 3 Oncological Pathology and Bioinformatics Research Group, Institut d’Investigació Sanitària Pere Virgili, Tortosa, Spain; 4 Department of Computer Engineering and Mathematics, Universitat Rovira i Virgili, Tarragona, Spain; 5 Research Unit, Hospital Universitario de Gran Canaria Dr. Negrín, Las Palmas de Gran Canaria, Spain; 6 Fundación Canaria Instituto de Investigación Sanitaria de Canarias (FIISC), Las Palmas de Gran Canaria, Spain; 7 Norwegian Institute of Food, Fisheries and Aquaculture Research (Nofima), Tromsø, Norway; 8 BCN MedTech, Department of Engineering, Universitat Pompeu Fabra, Barcelona, Spain

**Keywords:** Databases, Prognostic markers, Biophotonics, Biomedical engineering

## Abstract

Metastasis occurs in nearly 1 out of 3 breast cancer (BC) patients and significantly reduces survival rates, particularly in cases of distant metastases. As most distant metastases develop after diagnosis (i.e., recurrence) and remain incurable, there is a critical need for prognostic biomarkers to assess recurrence risk. Multimodal data analysis has emerged as a promising approach to integrate diverse information, offering a more comprehensive perspective. This study introduces the Histology HSI-BC (hyperspectral imaging - breast cancer) Recurrence Database, the first publicly accessible multimodal database designed to advance BC distant recurrence prediction. The database comprises 47 histopathological whole-slide images, 677 hyperspectral (HS) images, and clinical and demographic data from 47 BC patients, of whom 22 (47%) experienced distant recurrence over a 12-year follow-up. Histopathological slides were digitized using a whole-slide scanner and annotated by expert pathologists, while HS images were acquired with an HS camera coupled to a bright-field microscope. This database provides a promising resource for studying BC recurrence prediction and personalized treatment strategies by integrating the aforementioned multimodal data.

## Background & Summary

In 2022, breast cancer (BC) was the most common type of cancer in women, with an incidence of 23.8%, and the leading cause of cancer-related death among women, accounting for 15.4% of all cancer-related deaths^1^. Cancer cells can spread from the primary tumor to other parts of the body, which is known as metastasis and is the main cause of death in most cancers^2,3^. Metastasis occurs in nearly 1 out of 3 patients diagnosed with BC and can appear in the axillary lymph nodes (regional metastasis) or in other organs (distant metastasis)^4^. Overall, the 5-year survival rate after diagnosis of BC is 91%. However, this rate is higher in patients with tumors located exclusively in the breast (99%) than in patients with regional metastasis (86%) or with distant metastasis (31%), where survival decreases dramatically^4^. Women with distant metastases may have either *de novo* distant spread, where distant metastases are already present at the time of diagnosis, or develop distant metastases after an initial diagnosis and treatment, which is known as *recurrence*^5^. While de novo cases account for approximately 25% of metastatic BC diagnoses, the majority result from recurrence^6^. Whether de novo or recurrent, distant metastases remain incurable^7,8^.

Certain classic prognostic factors are associated with the risk of developing distant metastasis, such as *age*, *tumor diameter*, *stage*, *tumor grade*, *tumor type* or *lymphovascular invasion* (LVI)^8^. Additionally, studies have identified other biomarkers with prognostic value in the disease that may be associated with metastasis, including genetic alterations, circulating tumor cells and circulating tumor DNA, biomarkers of response to immunotherapy and gene expression platforms to predict the risk of recurrence^9–11^. However, to date there is no consensus for the implementation of most of these biomarkers in routine clinical practice. Therefore, there continues to be a growing interest in identifying specific prognostic biomarkers that allow determining the probability of developing metastasis.

Cancer detection relies heavily on imaging methods like X-ray, ultrasound, and magnetic resonance imaging^12^. However, treatment decisions require a conclusive histopathological diagnosis, which is obtained from a tissue biopsy. BC can be broadly categorized into in situ carcinoma and invasive carcinoma. Among these, ductal carcinoma in situ (DCIS) represents the most prevalent subtype of in situ carcinoma, while invasive ductal carcinoma (IDC) is the most common subtype of invasive carcinoma. Nevertheless, given the heterogeneity of BC, the accurate identification of these subtypes among other histological subtypes requires extensive expertise and a deep understanding of breast pathology^13^. The rise of digital pathology, which leverages whole-slide images (WSIs), has revolutionized research and diagnosis in pathology, particularly in cancer, by enabling more efficient data sharing across institutions and promoting remote collaborations. WSIs are high-resolution digital images of traditional glass pathology slides, which can be viewed, analyzed, and shared on a computer screen^14,15^. The use of WSIs also paves the way for computational pathology, which started from the use of traditional image analysis methods to advanced machine learning (ML) and deep learning (DL) algorithms^16,17^. Remarkably, these novel approaches offer the potential to integrate multiple data modalities, extending beyond histopathology image analysis. This includes linking histopathological images with clinical factors, such as prognosis and genetic mutations, thereby enhancing BC diagnostics^18–20^.

Beyond conventional methods, other imaging modalities show promising potential for improving the diagnosis and prognosis of BC patients. Among these, hyperspectral (HS) imaging (HSI), combines traditional imaging with spectroscopy to capture both spatial and spectral information. Each material interacts uniquely with emitted radiation, reflecting and absorbing it in a way that creates a distinct radiance vector, often named *spectral signature*. HSI sensors can capture these spectral signatures, acquiring significantly more data than standard RGB (Red, Green, Blue) cameras and extending imaging capabilities beyond human vision (e.g., near-infrared (NIR) HS sensors can capture wavelengths ranging from 900 to 1,700 nm)^21^. In recent years, the use of HSI in medicine has begun to achieve promising results regarding cancer detection by utilizing cutting-edge ML algorithms to process the high amount of HS data^22–24^. In the existing literature for medical histological applications, HSI has been used to identify pancreatic neoplasms with different prognoses^25^, quantify Ki67 as a prognostic factor in lymphomas^26^ and study the interactions between tumor cells and immune cells of the tumor microenvironment in response to immunotherapy in lung cancer^27^, obtaining promising results.

Research in this area is still in its early stages, and the number of published studies remains limited. Regarding WSI and clinical and demographic databases, The Cancer Genome Atlas (TCGA) is one of the main publicly available sources for hematoxylin and eosin (H&E)-stained WSIs and associated clinical and demographic data^28^. The main challenge of this database is the lack of annotations, which makes the subsequent analysis of these WSIs difficult. The Molecular Taxonomy of Breast Cancer International Consortium (METABRIC) is another database that provides clinical, demographic, and molecular data of over 2,000 BC cases^29^. However, it primarily focuses on genomic and transcriptomic data and does not include WSIs. Several publicly available databases contain H&E-stained WSIs^30^, but most include annotations focused on specific cell types in BC, such as tumor-infiltrating lymphocytes and inflammatory cells. We have identified one database - Breast Cancer Semantic Segmentation (BCSS) that provides specific annotations of tissue compartments, distinguishing between tumor and healthy tissue^31^. Regarding medical HS databases only two major sets were identified: in 2022, Zhang *et al*.^32^ introduced a large-scale database for HS microscopic images of precancerous lesions in gastric cancer, and in 2024, Ortega *et al*. released the HistologyHSI-GB dataset^33^, focused on HS glioblastoma histology. None of those databases included clinical or demographic data, this being one of the main challenges in this field. There is a limited availability of comprehensive, high-quality databases, which hinders the broader application of clinical and demographic data, WSIs and HSI in clinical practice and research. The lack of such databases makes it difficult to fully explore the potential of HSI together with conventional practices for diagnosing and predicting disease outcomes, such as recurrence in BC.

**Fig. 1:**
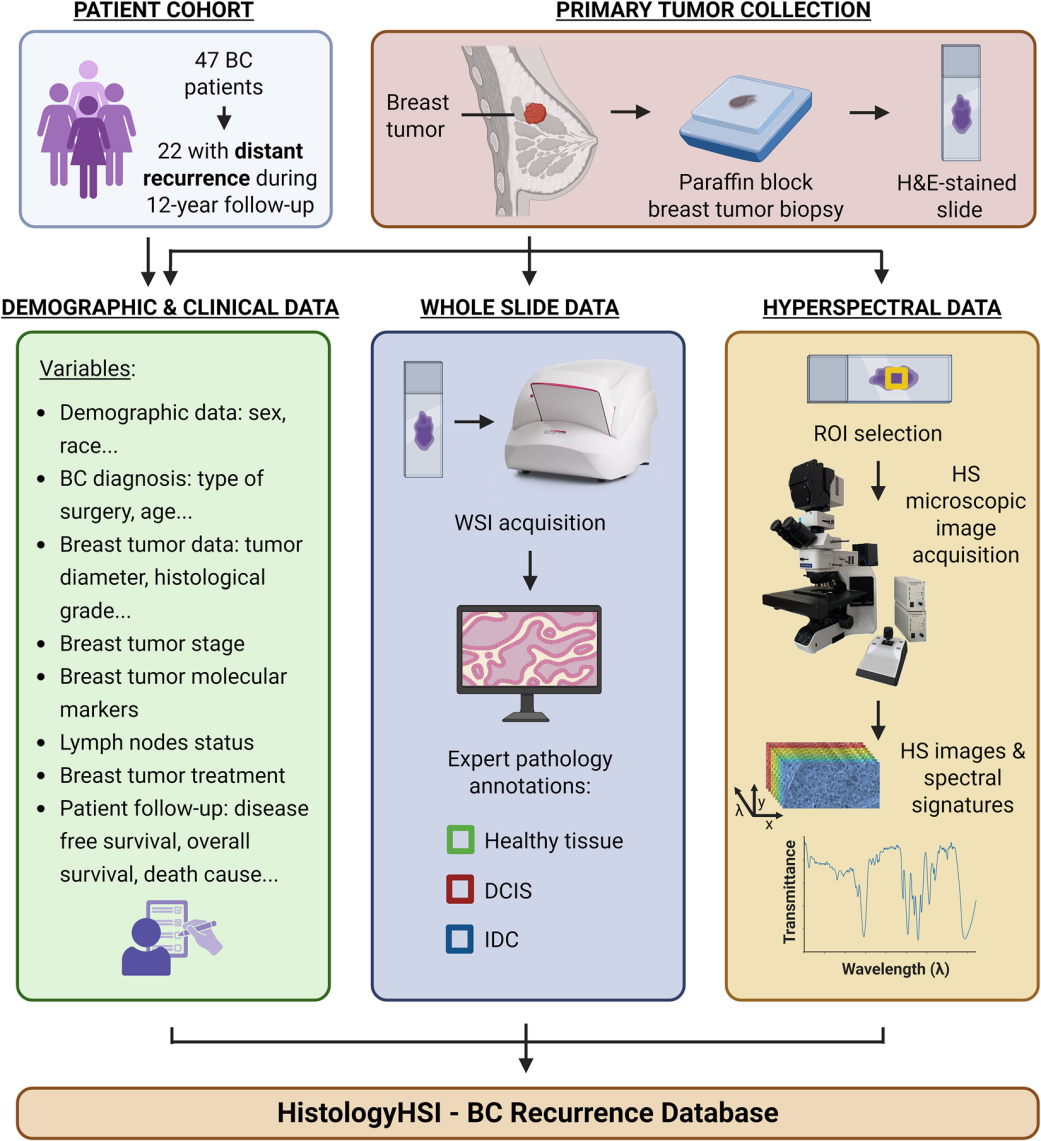
Schematic overview of the study design or workflow. The patient cohort comprises 47 BC patients, of which 22 (47%) experienced distant recurrence over a 12-year follow-up period and 25 (53%) did not. All these patients had a breast tumor biopsy, which was collected, prepared, and stained with H&E. The H&E-stained slides were digitized to obtain WSIs and annotated to differentiate three tissue compartments: IDC, healthy tissue and DCIS. Within each annotated area, ROIs were selected, from which HS images were acquired to generate HS cubes and extract spectral signatures. Together with the clinical and demographic data, all this information constitutes the HistologyHSI - BC Recurrence Database.

This paper presents a publicly accessible database designed to investigate specific prognostic biomarkers for predicting the likelihood of BC recurrence due to distant metastasis. The HistologyHSI-BC Recurrence Database includes clinical and demographic data from BC patients, along with WSIs and HS images obtained from their primary tumor samples. This database is intended to evaluate the ability to predict recurrence due to distant metastasis over a 12-year follow-up period. Biopsies from 47 patients diagnosed with BC were extracted, sliced and stained with H&E, 47 WSIs and 677 microscopic HS images were taken, and their clinical and demographic data were collected. Among these patients, 22 experienced distant recurrence. A schematic overview of the study workflow is presented in Fig. 1.

The HistologyHSI-BC Recurrence Database will benefit researchers by providing a comprehensive, multimodal database that integrates WSIs, HS images, and clinical and demographic data from BC patients. This resource enables the development and refinement of predictive models for BC recurrence due to distant metastasis, starting to fill a significant gap in available databases. Researchers can leverage this data to explore innovative ML approaches, enhance diagnostic accuracy, and identify novel biomarkers for BC recurrence. Additionally, the database promotes reproducibility, facilitates collaboration across institutions, and accelerates research in computational pathology, potentially improving personalized treatment strategies and benefiting broader cancer research efforts.

## Methods

### Patients selection, eligibility criteria and ethics approval

This is a retrospective case-control study carried out on 47 BC patients diagnosed with IDC, now called invasive breast carcinoma of no special type^34^, between 2006 and 2015, who met the eligibility criteria for inclusion (Table 1). Cases include 22 patients who experienced recurrence due to distant metastasis during the 12 years following diagnosis. The remaining 25 patients who did not experience recurrence during the 12 years of follow-up are included as control group.

**Table 1:** Eligibility criteria for patient inclusion.

Inclusion	Exclusion
A diagnosis of IDC	Receipt of neoadjuvant treatment, as it is known to modify the tumor microenvironment
Representativeness of IDC tissue in surgical biopsy	Recurrence occurring in the breast rather than in distant organs
A clinical history with complete clinical and pathological data	Presence of distant metastases at the time of diagnosis
Patient’s agreement to be included in the study	Failure to meet any of the inclusion criteria

The study was approved by the Drug Research Ethics Committee of the Institut d’Investigació Sanitària Pere Virgili (IISPV), Tarragona, Spain, under reference number 134/2022. The samples used in this study were obtained from Biobank IISPV-Node Tortosa, Tarragona, Spain, following the principles of ethical conduct and data protection. The Biobank has approved the open publication of the data associated with this work. All participants whose samples were stored in the biobank have previously signed an informed consent form, explicitly authorizing the collection, storage, and future use of their biological materials and associated data for research purposes. The processing, communication and transfer of personal data of all participants comply with the provisions of Organic Law 3/2018, of December 5, on the Protection of Personal Data and Guarantee of Digital Rights and with Regulation (EU) 2016/679 of the European Parliament and of the Council, of April 27, 2016, on the protection of natural persons with regard to the processing of personal data and the free circulation of these data, and repealing Directive 95/46/EC (General Data Protection Regulation). The data generated and collected during this study are anonymized to ensure the security of the information, safeguarding the confidentiality and privacy of the patients.

## Data Collection

### Clinical and demographic data

The data collection process involved extracting information from clinical records, including demographic and clinical data, which were following Table 2.

**Table 2:** Description of the study variables.

	Attribute	Definition	Format
**Demographic Data**	**Sex**	Patients’ gender	1: Female
	**Race**	Patients’ race	1: White
	**Ethnicity**	Patients’ ethnicity	1: Hispanic
	**Menopausal status**	Menopausal status of the patient	0: Premenopause and 1: Postmenopause
**Diagnosis**	**Dx surgery**	Type of surgery	0: Mastectomy and 1: Lumpectomy
	**Dx age**	Difference between diagnosis and birth dates	Years
**Tumor Data**	**Tumor diameter**	Maximum diameter of the irregular shaped tumor	Millimeters
	**Tumor histologic grade**	Degree of differentiation of tumor cells, reflecting how different they are from normal breast cells	1: Grade 1, 2: Grade 2 and 3: Grade 3
	**LVI**	Presence of tumor cells within lymphatic or blood vessels	0: Negative and 1: Positive
	**PNI**	Ability of cancer cells to proliferate around peripheral nerves and, eventually, invade them	0: Negative and 1: Positive
**Tumor Stage**	**T (tumor)**	Tumor size assessed by pathological evaluation	1: T1, 2: T2, 3: T3, and 4: T4
	**N (node)**	The cancer has spread to the LNs assessed by pathological evaluation	0: N0, 1: N1, 2: N2 and 3: N3
	**M (metastasis)**	Status of breast cancer spreading to a different part of the body	0: M0
**Tumor Molecular Markers**	**ER**	Status of ER	0: Negative and 1: Positive
	**PR**	Status of PR	0: Negative and 1: Positive
	**HER2**	Status of HER2	0: Negative and 1: Positive
	**KI67**	Index quantifying KI67 expression to measure how fast cancer cells are dividing in a tumor	0: KI67 index < 20% and 1: KI67 index ≥ 20%
	**Molecular subtype**	Classification according to IHC status of ER, PR, HER2 and Ki67	0: Luminal A, 1: Luminal B HER2-, 2: Luminal B HER2+, 3: HER2+, and 4: Triple negative
LN**s Status**	**LN status**	Status of the spreading of tumor cells to the SLNs and non-SLNs	0: Negative, 1: ITC, 2: Micrometastasis and 3: Macrometastasis
	**LN ITC number**	LNs with ITC	Natural number
	**LN MICRO number**	LNs with micrometastasis	Natural number
	**LN MACRO number**	LNs with macrometastasis	Natural number
	**LN number**	LNs removed during SLN biopsy and/or LN dissection	Natural number
	**SLN number**	LNs removed during SLN biopsy	Natural number
	**SLN status**	Presence (or absence) of tumor cells in the SLNs	0: Negative, 1: ITC, 2: Micrometastasis and 3: Macrometastasis
**Tumor Treatment**	**Tx hormonal**	Patient received (or not) hormonal treatment	0: Not received and 1: Received
	**Tx CT**	Patient received (or not) adjuvant CT after the surgery	0: Not received and 1: Received
	**Tx trastuzumab**	Patient received (or not) trastuzumab	0: Not received and 1: Received
	**Tx RT**	Patient received (or not) RT	0: Not received and 1: Received
**Follow-up**	**Metastasis type**	Status of cancer spreading from the primary tumor to other organs during the follow-up period	0: No evidence of local or distant metastases, 1: Metastasized on nearby tissues or LNs, 2: Metastasized in distant organs from primary site and 3: Both local and distant metastases are present
	**DFS**	Time a patient survives without any signs or symptoms of cancer after finishing primary treatment. Difference between the relapse and diagnosis dates. If the patient did not relapse, the date of last follow-up is used instead.	Months
	**Vital status**		0: Alive and 1: Deceased
	**Death cause**		0: Other causes / Still alive and 1: Cancer
	**OS**	Time from the date of cancer diagnosis that patients remain alive. Difference between the death and diagnosis dates. If the patient did not die, the date of last follow-up is used instead.	Months

CT, chemotherapy; DFS, disease-free survival; Dx, diagnosis; ER, estrogen receptors; HER2, human epidermal growth factor receptor 2; IHC, immunohistochemistry; ITC, isolated tumor cells; KI67, proliferation index; LN, lymph node; LVI, lymphovascular invasion; MACRO, macrometastasis; MICRO, micrometastasis; OS, overall survival; PNI, perineural invasion; PR, progesterone receptors; RT, radiotherapy; SLN, sentinel lymph node; Tx, treatment.

### Histopathology WSIs

Paraffin blocks of primary tumor biopsies with sufficient representative IDC tissue were obtained from the Biobank IISPV-Node Tortosa, Tarragona, Spain. The samples were processed in the Pathology Department, where 2 µm-thick sections were prepared from each paraffin block and stained with H&E according to the instructions of the manufacturer. The slides were sealed with coverslips using dibutylphthalate polystyrene xylene (DPX) mounting medium for subsequent digitization and HS microscopic image acquisition.

**Fig. 2:**
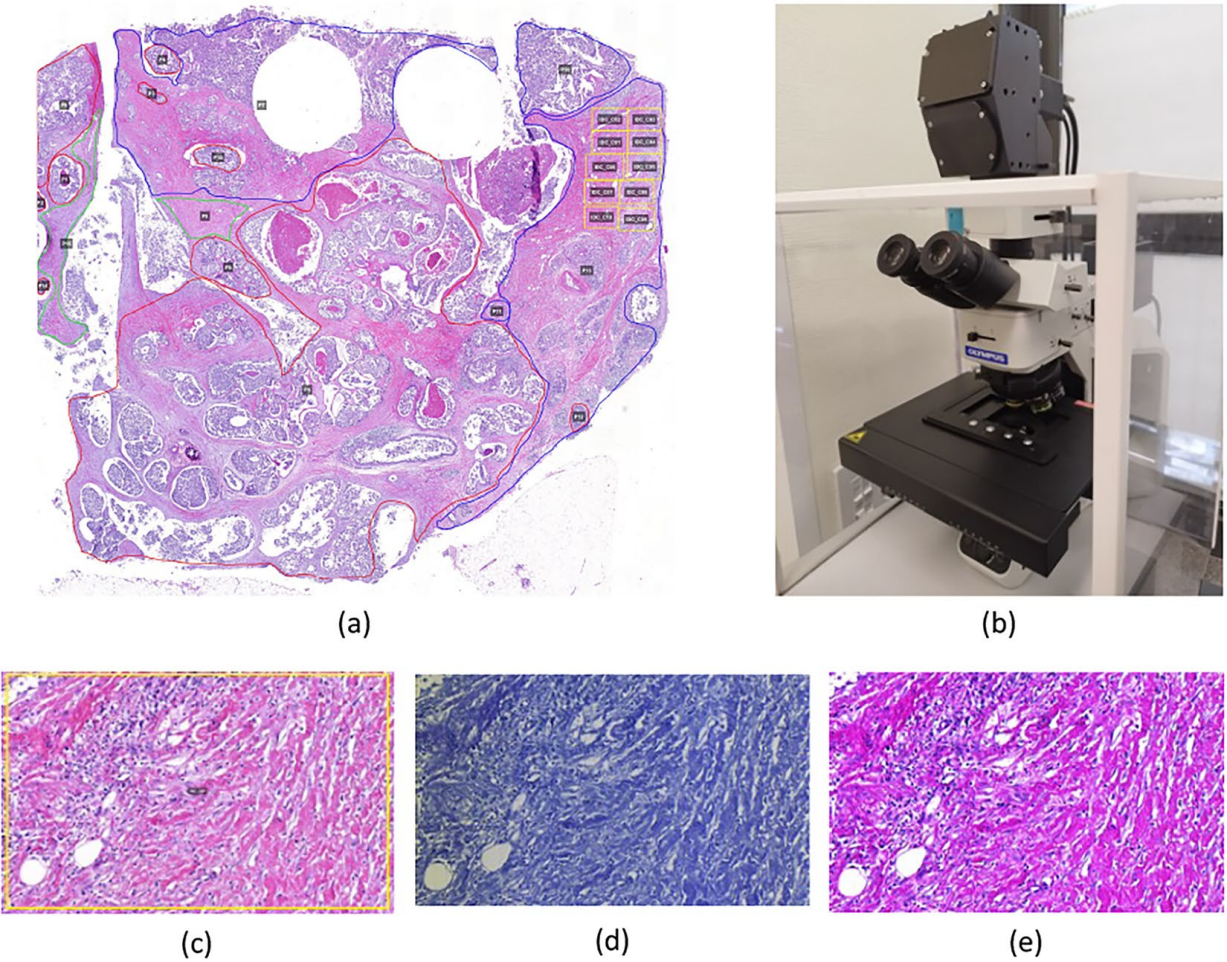
Elements and outputs to capture an HS image. (**a**) Annotated WSI (IDC outlined in blue, healthy tissue in green, and DCIS in red) captured with the WSI scanner. (**b**) HS microscopic system used to acquire an HS image and its corresponding high spatial resolution RGB image of a selected ROI. (**c**) Zoom-in of one of the selected ROIs outlined by yellow rectangles in a). (**d**) Synthetic RGB image generated from the captured HS image. (**e**) High spatial resolution RGB image captured using the 20 MP RGB camera of the HS microscopic system.

The H&E-stained slides were digitized with the Pannoramic 250 Flash III WSI scanner (3DHISTECH Ltd., Budapest, Hungary) at 20 × magnification (0.2433 µm/pixel) using MRXS image format. WSIs were visualized using QuPath^35^ (available at: https://qupath.github.io/) for technical validation and annotation, and anonymized using the SlideMaster software (3DHISTECH Ltd., Budapest, Hungary). The annotation process of each WSI was manually performed by pathologists using diverse colors to distinguish between IDC, healthy tissue, and DCIS. The annotations were made with the following color scheme: IDC was outlined in blue, healthy tissue in green, and DCIS in red (Fig. 2a).

Moreover, within each of these three tissue compartment types, different regions of interest (ROIs), surrounded by yellow line, were identified and annotated to subsequently acquire the HS image using the HS microscopic system (Fig. 2a). These ROIs were selected to ensure the inclusion of representative areas of each class (IDC, healthy and DCIS), capturing relevant spectral variability for further analysis.

### HS images

The HS images were captured using a HS microscopic system (Fig. 2b). The system features the Hyperspec® VNIR (Visible and Near Infrared) A-Series camera (HeadWall Photonics, Fitchburg, MA, USA), a pushbroom HS camera that captures data by scanning the sample spatially. The camera is equipped with a charge-coupled device (CCD) sensor that covers a spectral range of 400–1,000 nm, capturing 826 spectral bands across 1,004 spatial pixels per line. It offers high spectral resolution with a slit image full width at half maximum (FWHM) of 2.5 nm and a pixel size of 7.4 μm. Data are acquired with a 12-bit ADC (Analog-to-digital Converter), and each HS line has a size of 1,004 × 826 pixels and requires 1.6 MB per line on disk for storage. The microscope used is the OLYMPUS BX-53 (Olympus, Tokyo, Japan), with LMPLN-IR (5 × , 10 × ) and LCPLN-IR (20 × , 50 × ) objective lenses optimized for infrared imaging. The system uses a 100 W TH4 halogen lamp (Olympus, Tokyo, Japan) as the light source, covering a wavelength range from 400 to 1,800 nm and supporting both transmittance and reflectance light paths. To acquire full HS images, the pushbroom camera requires spatial scanning, which is facilitated by a SCAN 130 × 85 scanning stage (Märzhäuser, Wetzlar, Germany). The stage ensures high precision ( ± 3 μm accuracy) as it moves the sample, keeping it aligned with the objective and light path. Furthermore, an RGB camera, the acA5472-17uc (Basler AG, Ahrensburg, Germany), provides real-time visualization of the sample to navigate it without the need of using the microscope binoculars. It is a 20 MP compact camera with a Sony IMX183 CMOS sensor (Tokyo, Japan), 5,496 × 3,672 resolution, and 17 fps. It features USB 3.0, a C-mount, and supports hardware/software triggers.

Calibration of the HS images is necessary to ensure the data accurately represents the sample’s spectral signatures. The HS microscope captures spectral signatures for each pixel, but factors like the sensor’s response, light transmission, and the light source can affect accuracy. The calibration process involves normalizing the pixel values of the HS image by adjusting them based on a white reference (WR) and a dark reference (DR). WR is obtained by focusing on an empty area of the slide at the same working distance. This ensures no sample material is present, allowing the frame to record the maximum signal the sensor can measure for each pixel and wavelength under the given conditions (e.g., exposure time, light intensity, and slide properties). Conversely, the DR is captured by completely blocking light transmission to the HS camera. This frame captures the minimum signal levels detectable by the sensor for each pixel and band, as well as dark current information from the CCD. Ideally, DR values approach zero; however, higher values may occur due to intrinsic sensor noise. To enhance the reliability of the calibration process, 100 frames are collected for both the WR and DR, ensuring that averaging reduces potential errors. Finally, the calibration of the HS image is achieved using Eq. (1), which relates the calibrated HS image (${r}_{i}$) to the raw HS image (${{Raw}}_{i}$).

1 $${r}_{i}=\frac{{{Raw}}_{i}-{DR}}{{WR}-{DR}}$$

In-house software was developed to serve multiple functions in the HSI acquisition process. It displays the RGB image to facilitate sample navigation under the microscope and ensures synchronization between the HS camera and the scanning platform by aligning their frame rate and platform movement. After capturing the HS image, the software removes the extreme bands from the raw HS image (reducing the spectral range from 400–1,000 nm to 400.5–938 nm), as these bands contain significant noise, and then saves the raw HS image. The calibrated HS image is then generated, using Eq. (1), and saved on memory as five-digit 16-bit unsigned integers (uint16), where the most significant digit represents the units, and the remaining digits correspond to the decimal places of the transmittance values. Therefore, to obtain true transmittance values, the calibrated HS image must be divided by 10^4^. Additionally, the software creates a synthetic RGB image, following the methodology explained by Ortega *et al*.^33^, to facilitate the visualization of the spatial characteristics of the HS image.

Prior to any HS image capture, magnification is selected, in this case the 10×. WR and DR reference images are collected. Then, to acquire the HS image, the associated WSI is examined in QuPath^35^ to identify an ROI within a specific class, such as IDC (blue), healthy (green) or DCIS (red) tissue. The identified ROI is searched for in the HS microscopic system using the RGB camera and marked down on the histological image using a yellow rectangle (Fig. 2c). The ROI is then captured using the HS microscope, generating the raw HS image, the calibrated HS image, and the synthetic RGB image (Fig. 2d). The RGB image of the ROI is also captured (Fig. 2e) for future analysis. All data corresponding to one of these captures are saved using an identifier with its corresponding metadata, including the patient identifier, classification, and region (e.g., HSI_VNIR_15_IDC_x10_C01; see Data records section for more details).

## Data Records

**Fig. 3:**
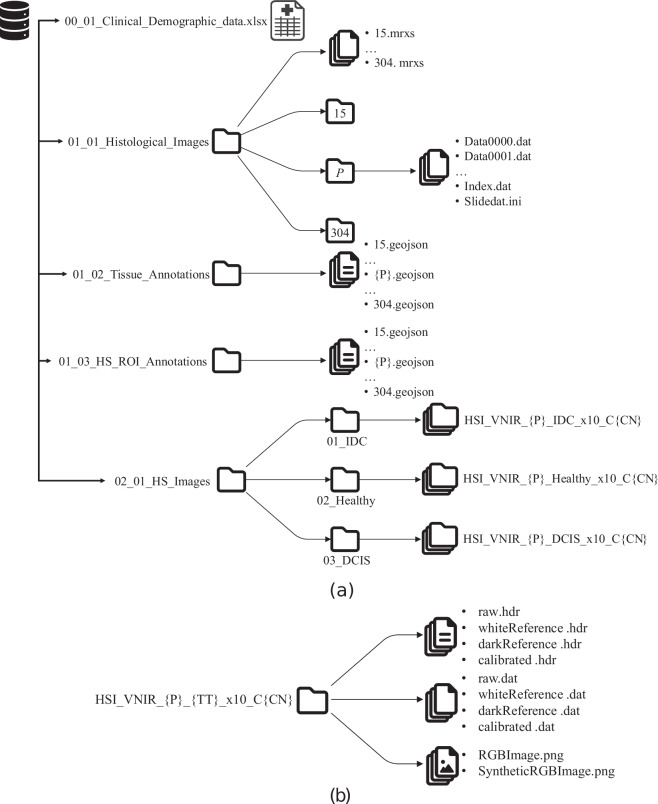
Graphical representation of (**a**) the HistologyHSI-BC Recurrence Database structure and (**b**) the contents of each HS image capture.

The HistologyHSI-BC Recurrence Database^36^ has been deposited at The Cancer Imaging Archive (TCIA) repository^37^ to make it publicly available, organized into a multilayer folder arrangement. The database is divided into three main components: clinical and demographic data, histological WSI and HS images (see Fig. 3a). The clinical and demographic data are stored at the *00_01_Clinical_Demographic_Data* file. This XLSX file documents patients’ demographic status, breast tumor characteristics, treatment received, and their follow-up outcomes (detailed description on Table 2).

Furthermore, the histological data are structured in 3 folders. Firstly, *01_01_Histological_Images* folder contains the WSI for each patient, stored as MRXS files. Each WSI requires a corresponding metadata folder containing DAT and INI files for proper rendering. Moreover, *01_02_Tissue_Annotations* folder includes WSI histological annotations that classify tissue types, with boundaries of the regions outlined in blue (IDC), green (healthy), and red (DCIS), as shown in Fig. 2a. The third folder, *01_03_HSI_ROI_Annotations* contains the ROI for each HS image, with the boundaries of the region outlined in yellow (Fig. 2a). Both histological WSI and HS image ROI annotation files are provided in GeoJSON format. A summary of the areas of annotations per patient and tissue type is shown in Table 3. Lastly, *02_01_HSI_Images* folder contains the HS images of the histological slides, stored in ENVI format^38^. Each capture includes the raw HS image, WR and DR calibration files, and the calibrated HS image following the procedure described in Eq. (1). As the ENVI standard states, the HS image is saved as a flat-binary raster DAT (data) file with an accompanying HDR (header) file containing essential metadata to interpret it. Moreover, within each capture folder a synthetic RGB image and a view of the ROI captured by the RGB camera are stored. The HS image data are stored in folders named according to the regular expression *HSI_VNIR_{P}_{TT}_x10_C{CN}*, where {P} represents the patient ID, {TT} indicates the tissue type (IDC, healthy, or DCIS), and {CN} is the capture number (Fig. 3b).

**Table 3:** Summary of histological area coverage and capture counts by tissue type per image.

Patient ID	Tissue Annotations Area [mm^2^]			HS image ROI Annotations [Number of captures]			Recurrence Label
	IDC	Healthy	DCIS	IDC	Healthy	DCIS	
**15**	40.94	2.92	64.24	10	0	0	0
**19**	94.89	24.00	0.00	10	5	0	1
**20**	169.27	58.36	0.28	10	5	0	1
**25**	135.97	139.29	17.62	10	5	5	0
**38**	210.59	7.71	0.23	10	5	0	1
**40**	72.19	20.77	0.00	10	5	0	1
**43**	57.46	22.70	0.00	10	5	0	0
**45**	71.77	80.06	6.70	10	5	5	0
**47**	10.40	137.41	0.00	10	5	0	0
**51**	163.12	2.79	2.34	10	5	0	0
**52**	96.22	3.50	0.00	10	3	0	0
**57**	23.26	31.54	0.00	10	5	0	0
**62**	4.40	66.22	0.00	8	5	0	0
**65**	104.57	15.22	0.00	10	5	0	0
**68**	21.41	75.70	0.00	10	5	0	0
**70**	55.12	45.12	0.00	10	5	0	0
**80**	4.84	37.06	0.00	7	5	0	0
**82**	109.32	11.99	0.31	10	5	0	0
**84**	88.06	13.20	7.50	10	5	0	1
**85**	119.25	5.21	20.59	10	5	5	0
**90**	179.57	29.70	0.00	10	5	0	0
**100**	68.16	3.21	0.06	9	4	0	1
**107**	8.43	192.86	7.70	10	5	5	0
**112**	14.84	1.68	0.00	10	0	0	0
**124**	26.84	0.36	0.00	10	0	0	0
**136**	190.00	25.98	28.52	9	4	5	0
**138**	32.58	90.49	11.91	10	5	0	0
**139**	43.23	72.05	0.00	10	5	0	0
**141**	168.53	7.96	5.27	10	5	0	1
**146**	24.16	3.47	0.32	10	0	0	0
**151**	3.98	3.78	1.10	4	0	0	0
**152**	82.41	42.77	19.22	10	5	5	1
**153**	7.06	77.57	0.88	8	5	0	0
**154**	51.74	0.00	0.00	10	0	0	1
**162**	67.66	16.19	1.72	10	5	0	1
**189**	247.15	0.02	0.00	10	0	0	1
**197**	212.83	57.60	4.90	10	5	5	1
**205**	321.29	35.69	0.14	10	5	0	1
**211**	161.00	0.10	0.09	10	0	0	1
**213**	451.91	7.64	0.00	10	5	0	1
**229**	281.13	3.09	2.02	10	3	0	1
**238**	149.90	0.00	0.00	9	0	0	1
**255**	164.85	35.81	0.50	10	5	0	1
**259**	92.43	42.47	2.35	10	5	0	1
**269**	59.97	6.65	0.00	10	5	0	1
**270**	30.11	111.68	0.00	10	5	0	1
**304**	155.02	24.54	0.00	10	5	0	1

## Technical Validation

### Clinical and demographic data statistic analysis

A preliminary statistical analysis was conducted to identify differences in the variables between patients with and without recurrence, as shown in Table 4. Statistical tests used for comparisons included the absolute frequency (percentage) for the Chi-square test or Fisher’s exact test, and the median (interquartile range) for the Mann-Whitney U test. As expected, certain classic clinical and pathological variables were found to be associated with the risk of developing metastasis in the present cohort^8^, including age at diagnosis, tumor diameter, and LVI.

**Table 4:** Differences in the clinical and demographic variables in recurrence vs. non-recurrence groups.

	Attribute	Format	Recurrence	Non-Recurrence	*p*
**Demographic Data**	**Sex**	1: Female	22 (100.0)	25 (100.0)	—
	**Race**	1: White	22 (100.0)	25 (100.0)	—
	**Ethnicity**	1: Hispanic	22 (100.0)	25 (100.0)	—
	**Menopausal status**	0: Premenopause 1: Postmenopause	3 (13.6) 19 (86.4)	3 (12.0) 22 (88.0)	1.000*
**Diagnosis**	**Dx surgery**	0: Mastectomy 1: Lumpectomy	7 (31.8) 15 (68.2)	2 (8.0) 23 (92.0)	0.063*
	**Dx age**	Years	73.0 [22.0]	57.0 [14.0]	**0.017**^**‡**^
**Tumor Data**	**Tumor diameter**	Millimeters	26.5 [13.8]	15.0 [13.0]	**<0.001**^**‡**^
	**Tumor histologic grade**	1: Grade 1 2: Grade 2 3: Grade 3	1 (4.5) 11 (50.0) 10 (45.5)	4 (16.0) 14 (56.0) 7 (28.0)	0.285*
	**LVI**	0: Negative 1: Positive	8 (36.4) 14 (63.6)	18 (72.0) 7 (28.0)	0.031*
	**PNI**	0: Negative 1: Positive	15 (68.2) 7 (31.8)	21 (84.0) 4 (16.0)	0.351*
**Tumor Stage**	**T (tumor)**	1: T1 2: T2 3: T3 4: T4	5 (22.7) 14 (63.6) 2 (9.1) 1 (4.5)	17 (68.0) 8 (32.0) 0 (0.0) 0 (0.0)	0.012*
	**N (node)**	0: N0 1: N1 2: N2 3: N3	8 (36.4) 7 (31.8) 5 (22.7) 2 (9.1)	25 (100.0) 0 (0.0) 0 (0.0) 0 (0.0)	<0.001*
	**M (metastasis)**	0: M0	22 (100.0)	25 (100.0)	—
**Tumor Molecular Markers**	**ER**	0: Negative 1: Positive	4 (18.2) 18 (81.8)	5 (20.0) 20 (80.0)	1.000*
	**PR**	0: Negative 1: Positive	6 (27.3) 16 (72.7)	9 (36.0) 16 (64.0)	0.744*
	**HER2**	0: Negative 1: Positive	15 (68.2) 7 (31.8)	22 (88.0) 3 (12.0)	0.154*
	**KI67**	0: KI67 index < 20% 1: KI67 index ≥ 20%	4 (18.2) 18 (81.8)	10 (40.0) 15 (60.0)	0.189*
	**Molecular subtype**	0: Luminal A 1: Luminal B HER2− 2: Luminal B HER2+ 3: HER2+ 4: Triple negative	4 (18.2) 10 (45.5) 4 (18.2) 3 (13.6) 1 (4.5)	6 (24.0) 13 (52.0) 2 (8.0) 1 (4.0) 3 (12.0)	0.512*
**LNs Status**	**LN status**	0: Negative 1: ITC 2: Micrometastasis 3: Macrometastasis	6 (27.3) 2 (9.1) 3 (13.6) 11 (50.0)	22 (88.0) 3 (12.0) 0 (0.0) 0 (0.0)	<0.001*
	**LN ITC number**	Number of LNs with ITC	0.0 [0.0]	0.0 [0.0]	0.720^‡^
	**LN MICRO number**	Number of LNs with micrometastasis	0.0 [0.0]	0.0 [0.0]	**0.027**^**‡**^
	**LN MACRO number**	Number of LNs with macrometastasis	0.5 [7.0]	0.0 [0.0]	**<0.001**^**‡**^
	**LN number**	Total number of LNs removed during SLN biopsy and/or LN dissection	13.0 [15.0]	2.0 [2.0]	**0.001**^**‡**^
	**SLN number**	Number of LNs removed during SLN biopsy	0.5 [2.0]	2.0 [2.0]	**<0.001**^**‡**^
	**SLN status**	0: Negative 1: ITC 2: Micrometastasis 3: Macrometastasis	4 (36.4) 2 (18.2) 2 (18.2) 3 (27.3)	22 (88.0) 3 (12.0) 0 (0.0) 0 (0.0)	0.002*
**Tumor Treatment**	**Tx hormonal**	0: Not received 1: Received	5 (22.7) 17 (77.3)	4 (16.0) 21 (84.0)	0.715*
	**Tx CT**	0: Not received 1: Received	11 (50.0) 11 (50.0)	14 (56.0) 11 (44.0)	0.906*
	**Tx trastuzumab**	0: Not received 1: Received	19 (86.4) 3 (13.6)	23 (92.0) 2 (8.0)	0.654*
	**Tx RT**	0: Not received 1: Received	3 (13.6) 19 (86.4)	3 (12.0) 22 (88.0)	1.000*
**Follow-up**	**Metastasis type**	0: No evidence of local or distant metastases 1: Metastasized on nearby tissues or LNs 2: Metastasized in distant organs from primary site 3: Both local and distant metastases are present	0 (0.0) 0 (0.0) 20 (90.9) 2 (9.1)	25 (100.0) 0 (0.0) 0 (0.0) 0 (0.0)	<0.001*
	**DFS**	Months	39.0 [48.0]	150.0 [28.0]	**<0.001**^**‡**^
	**Vital status**	0: Alive 1: Deceased	2 (9.1) 20 (90.9)	21 (84.0) 4 (16.0)	<0.001*
	**Death cause**	0: Other causes / Still alive 1: Cancer	5 (22.7) 17 (77.3)	25 (100.0) 0 (0.0)	<0.001*
	**OS**	Months	66.5 [85.0]	150.0 [28.0]	**<0.001**^**‡**^

Data are expressed as absolute frequency (percentage) for qualitative variables, compared using the Chi-square test or Fisher’s exact text*, and as median [interquartile range] for quantitative variables, analyzed using the Mann-Whitney U test^‡^.

CT, chemotherapy; DFS, disease-free survival; Dx, diagnosis; ER, estrogen receptors; HER2, human epidermal growth factor receptor 2; ITC, isolated tumor cells; KI67, proliferation index; LN, lymph node; LVI, lymphovascular invasion; MACRO, macrometastasis; MICRO, micrometastasis; OS, overall survival; PNI, perineural invasion; PR, progesterone receptors; RT, radiotherapy; SLN, sentinel lymph node; Tx, treatment.

Analysis of lymph nodes status revealed a significantly higher percentage of micrometastasis and macrometastasis in patients with recurrence compared to those without recurrence. This association remained significant when considering the number of affected lymph nodes. A similar trend was observed in sentinel lymph nodes status, where micrometastasis and macrometastasis were more prevalent in the recurrence group. However, no significant differences were found in lymph nodes containing isolated tumor cells, classified as negative lymph nodes^39^. These findings align with established knowledge that lymph node metastasis is associated with a higher risk of recurrence in BC patients during follow-up^5,40^.

Regarding patient follow-up, we confirmed that all patients without recurrence show no evidence of local or distant metastases, whereas patients with recurrence do, with most of them having metastases only in distant organs and a smaller percentage presenting with both local and distant metastases. Among patients with recurrence, 90.9% died, with cancer being the cause of death in 77.3% of cases. In contrast, among the patients without recurrence who died, none died from cancer. As expected, the median disease-free survival (DFS) was significantly shorter in the recurrence group compared to the non-recurrence group, as was overall survival (OS).

### Histopathology WSIs and annotation validation

**Fig. 4:**
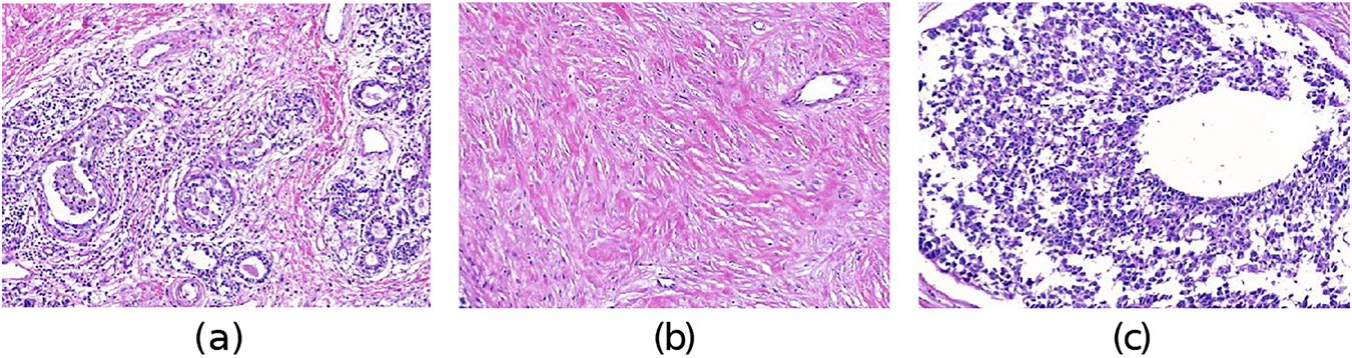
Examples of (**a**) IDC, (**b**) healthy, and (**c**) DCIS tissue types on a WSI at 2x magnification.

Pathologists qualitatively verified the quality of histopathological slides after the sectioning, processing, and staining phases. They confirmed the absence of artifacts in the ROIs of the WSIs, ensuring that these were not introduced during the tissue preparation or digitization phases. Pathologists did not detect the presence of folds, broken tissues, tears, bubbles, scalpel marks, or bad staining on the ROIs due to the tissue preparation phase. Furthermore, they verified the quality of the digitized histopathological slides making sure there were no issues on the WSIs due to the scanning phase. They confirmed the absence of scanning artifacts like focus issues or white reference problems. The annotations on the WSIs (IDC, healthy, and DCIS) were initially made by one pathologist, with the annotation process then subjected to validation by a second pathologist through a pairwise review. This validation phase played a crucial role in minimizing the inter-observability issue, ensuring consistency and reliability in the annotations. An example of the validated annotations is shown in Fig. 4.

### HSI validation

A technical validation was performed to ensure the quality of the HistologyHSI-BC Recurrence Database. The HS microscope employed in this study has been thoroughly characterized in previous works^41^, confirming its strong performance for spectral resolution-intensive applications. The system demonstrates a dynamic range of 65.3 ± 0.1 dB in transmittance mode, with a constant dark current of 20 digital numbers, which contributes to a reliable HS image capture. It is capable of capturing 826 spectral bands, providing detailed spectral information and accurately reflecting the spectral properties of the materials under analysis. This is evidenced by a spectral correlation measure of 0.88 ± 0.01 when capturing the WCT-2065 transmittance wavelength calibration standard (Avian Technologies, New London, USA) with a known spectral signature in transmittance mode. The system offers a spatial resolution of 0.739 ± 0.001 µm/pixel, along with a modulation transfer function (MTF) of 370 ± 10 line pairs/mm, ensuring sufficient detail for microscopic imaging. Spatial scanning accuracy is indicated by an eccentricity of 0.04 ± 0.04, and spatial repeatability is shown to have a relative difference of 14 ± 8% across consecutive captures. All values were measured at 10× magnification, the same magnification used for the HS image capturing process in this work.

**Fig. 5:**
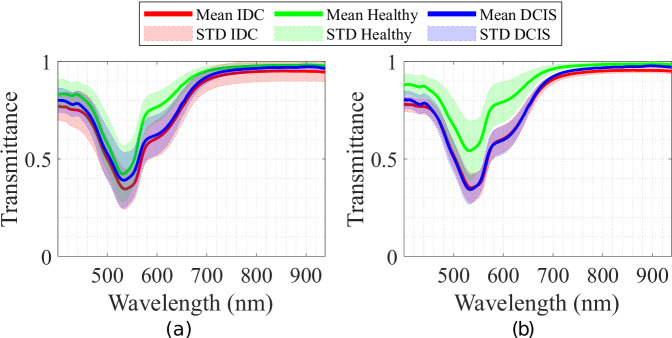
Mean and standard deviation HS spectral signatures for different tissues (IDC, healthy and DCIS) for patients (**a**) without and (**b**) with recurrence.

The characterization parameters obtained from the HS microscopic system demonstrate its capability to provide reliable and accurate HS data. The HS images captured from the 47 patients studied underwent a calibration. Afterwards, the database was evaluated to ensure the quality of the captured data. All HS images from each patient and tissue class (IDC, healthy and DCIS) were averaged for visualization purposes. Figure 5 groups the spectral signatures of patients with and without recurrence after 12 years. Interestingly, in the biopsies, patients without recurrence showed a greater similarity between healthy and DCIS tissues, while these tissue types were more distinctly separated in patients with recurrence. This finding raises the possibility that the closer resemblance of DCIS to healthy tissue could serve as an indicator of non-recurrence.

## Usage Notes

### Visualizing histopathology WSIs

The authors recommend downloading and installing the QuPath software^35^ to visualize the WSIs (MRXS format) and their related annotations (GeoJSON format) (Fig. 2a). Further image analysis can be performed using Python scripts (see sections Recommended histopathology WSI processing and Code availability). There are two ways to open a WSI in QuPath: drag and drop the MRXS file into QuPath or go on “File/Open” and select and open the MRXS file. There is also a tab on the left side of QuPath’s user interface called “Image”, in which it is possible to visualize the metadata of the histopathological image, such as width, height, magnification, and resolution. After opening the WSI on QuPath, the two available GeoJSON files containing annotations on the WSI should be imported. One includes the annotations related to the tissue compartments (IDC in red, healthy in green, and DCIS in blue). In contrast, the other defines the ROIs used for capturing the HS images, represented as yellow rectangles. These two files can be opened by dragging and dropping them into QuPath or clicking “File/Import objects from file” and selecting the GeoJSON files. The data from the GeoJSON files is visible by clicking on the tab “Annotations”. If the annotation classes are not shown after clicking the “Annotations” tab, click on the button with the three vertical dots on the bottom right of the tab panel, then select “Populate from existing objects/All classes (including sub-classes)” and the class types along with the number of annotations for each will appear.

### Recommended histopathology WSI processing

This section provides guidelines for working with WSIs (MRXS format), for which the use of Python scripts is recommended (see the Code availability section for more details). Due to their high resolution, efficient processing techniques are necessary to optimize performance and memory usage. Processing high-resolution images can be time-consuming and memory intensive. The highest available resolution of the selected slide image is approximately 85,000 × 202,000 pixels, making it significantly large. To optimize performance, a lower resolution (approximately 670 × 1,600) level should be selected for visualization. It is also important to downscale the annotations to match the selected lower resolution level.

### Recommended HSI processing

After HS data capture, the calibration of HS images is a mandatory step; however, additional processing may be performed depending on the specific application of the data.

- Given the strong correlation between adjacent spectral bands, spectral dimensionality reduction can be beneficial in reducing intrinsic Gaussian noise and computational costs. This can be accomplished by averaging adjacent spectral bands to create a spectrally reduced HS image. For example, the data could be reduced from the original 826 bands to 275 using a spectral window that includes three neighboring bands.
- Normalization is also recommended when partial absorbance is less critical, but the specific absorption wavelengths are significant. This normalization can be performed to scale the data between 0 and 1 or to have a mean of 0 and a standard deviation of 1.
- For HS analysis of the samples, it is advised to remove the sample background by identifying areas with no absorbance, typically represented by the white background.
- The classification of the data can be based on recurrence status and/or tissue type (IDC, healthy, or DCIS). When using ML or DL, it is crucial to ensure that data from the same patient do not appear simultaneously in the training, testing, or validation sets.

All these processing steps can be implemented using Python (see the Code availability section for further details).

### How to combine the different databases (clinical and demographic, WSIs, and HSI)

Integrating the diverse databases within the Histology HSI-BC Recurrence Database can enhance the prediction of distant recurrence in BC by leveraging complementary information from multiple modalities. Histopathological WSIs provide morphological insights assessed by pathologists, HS images capture biochemical variations that may indicate early tumor progression, and clinical and demographic data offer critical patient-specific factors. By integrating these databases, researchers can develop more robust predictive models that go beyond traditional histological or clinical assessments, improving risk stratification and supporting personalized treatment decisions.

Researchers can employ various data fusion strategies to achieve this integration. *Early fusion* involves combining raw or preprocessed features from each modality before model training, allowing the model to learn directly from the integrated data^42^. *Intermediate fusion* entails extracting high-level features from each database separately and then merging them into a joint representation, capturing modality-specific patterns prior to integration^43^. *Late fusion* consists of training independent models for each modality and subsequently combining their outputs to improve overall prediction accuracy^19^. Implementing these fusion techniques requires meticulous preprocessing to ensure compatibility and maximize the value of each database. By effectively integrating these multimodal databases, researchers can uncover subtle patterns associated with BC recurrence, advancing precision oncology and personalized patient care.

## Data Availability

The HistologyHSI-BC Recurrence Database is publicly available on the TCIA repository at 10.7937/6KPY-YT49.

## Code availability

The Python scripts developed for this study, along with the required Python toolboxes and libraries, are available in the publicly accessible repository at https://github.com/HIRIS-Lab/HistologyHSI-BC-Recurrence. The repository includes a main script providing a basic example of how to load and perform preliminary preprocessing of hyperspectral data in ENVI format using Python, for which the spectral library is required. In addition, the repository contains a tutorial demonstrating how to manipulate WSI in MRXS format and annotation in GeoJSON format in Python. The script overlays tissue compartments (IDC, healthy, and DCIS) onto slide images using their corresponding colors: blue, green, and red. Working with WSIs in Python requires the installation of the OpenSlide library. In addition, the JSON library is used for loading annotations, and the PIL library is employed to prepare and save slide images.
